# A prospective validation study in South-West Nigeria on caregiver report of childhood pneumonia and antibiotic treatment using Demographic and Health Survey (DHS) and Multiple Indicator Cluster Survey (MICS) questions

**DOI:** 10.7189/jogh.08-020806

**Published:** 2018-12

**Authors:** Adejumoke I Ayede, Amir Kirolos, Kayode R Fowobaje, Linda J Williams, Ayobami A Bakare, Oladapo B Oyewole, Oluwaseun B Olorunfemi, Oluwaseun Kuna, Nkechi T Iwuala, Abolanle Oguntoye, Simeon O Kusoro, Mofeyisade E Okunlola, Shamim A Qazi, Harish Nair, Adegoke G Falade, Harry Campbell

**Affiliations:** 1 Department of Paediatrics, College of Medicine, University of Ibadan, Ibadan, Nigeria; 2 University College Hospital, Ibadan, Nigeria; 3 Centre for Global Health Research, Usher Institute of Population Health Sciences and Informatics, University of Edinburgh, Edinburgh, Scotland, UK; 4 Department of Maternal Newborn Child and Adolescent Health, World Health Organization, Geneva, Switzerland; * Joint first authorships; † Joint last authorships

## Abstract

**Background:**

Childhood pneumonia is the single largest infectious cause of death in children under five worldwide. Demographic and Health Surveys (DHS) and Multiple Indicator Cluster Surveys (MICS) provide health information on care sought for sick children in resource poor settings. Despite not being primarily designed to identify childhood pneumonia, there are concerns that reported episodes of “symptoms of acute respiratory infection” in DHS and MICS are often interpreted by other groups as a “proxy” for childhood pneumonia. Using DHS5 and MICS5 survey tools, this study aimed to assess how accurately caregivers report of “symptoms of acute respiratory infection” reflect pneumonia episodes and antibiotic use in children under five.

**Methods:**

Children aged 0 to 59 months presenting with cough and/or difficult breathing were recruited from four study hospitals in Ibadan, Nigeria from August 2015 to March 2017. Children were assessed using World Health Organization (WHO) standard criteria by study physicians to identify whether they had pneumonia. Three hundred and two matched children in each category of ‘pneumonia’ and “no pneumonia” were followed up at home, either two or eight weeks later, using questions from DHS5 and MICS5 surveys to assess the accuracy of caregiver recall of pneumonia.

**Results:**

The specificity of DHS5 and MICS5 questions for identifying childhood pneumonia were 87.4 (95% confidence interval (CI) = 83.1-91.0) and 86.1 (95% CI = 81.7–89.8) respectively and the sensitivity of questions were 37.1 (95% CI = 31.6-42.8) and 37.1 (95% CI = 31.6-42.8). Correct recall of antibiotic treatment was poor (kappa statistic = 0.064) but improved with the use of medicine pill boards (kappa statistic = 0.235).

**Conclusions:**

DHS5 and MICS5 survey questions are not designed to identify childhood pneumonia and this study confirms that they do not accurately discern episodes of childhood pneumonia from cough/cold in children under five. The proportion of pneumonia episodes appropriately treated with antibiotics cannot be accurately assessed using current DHS and MICS surveys. If these results are used to guide programmatic decisions, it is likely to encourage overuse and inappropriate prescribing of antibiotics for episodes of cough/cold. International agencies who continue to use these household data to monitor the proportion of children with pneumonia who receive antibiotic treatment should be discouraged from doing this as these data are likely to mislead national and global programmes. Medicine pill boards are used in a number of DHS surveys and should be promoted for wider use in national population surveys to improve the accuracy of antibiotic recall.

Childhood pneumonia is the single largest infectious cause of death in children worldwide with an estimated 0.92 [0.812 − 1.117] million deaths in children under five in 2015 [1], equating to about 16% of all child deaths globally [2,3]. The majority of pneumonia deaths in children under five occur in low and lower-middle income countries in Sub-Saharan Africa and South-East Asia [2]. The goal of the integrated Global Action Plan for the Prevention and Control of Pneumonia and Diarrhoea (GAPPD) is to reduce deaths from pneumonia to fewer than 3 per 1000 live births in children under five by 2025 [4]. This involves improving care seeking for children with symptoms of pneumonia, access to health care, treatment with appropriate antibiotics where needed, coverage of pneumococcal vaccination and improvement of general health & well-being of children in low and lower-middle income countries. Monitoring trends in the proportion of childhood pneumonia cases correctly diagnosed and treated with antibiotics is therefore a vital element in the planning and monitoring of programmes aimed at controlling childhood pneumonia.

Demographic and Health Surveys (DHS) and Multiple Indicator Cluster Surveys (MICS) are household surveys conducted in low and lower-middle income countries every 3-5 years [5,6]. They collect information on health and well-being using standardized methods and tools. These are usually conducted on households chosen via a multi-stage cluster sampling design to provide a representative sample of the country being investigated. DHS and MICS outputs are used for many purposes, including supporting the monitoring of population health and health services. These data are particularly valuable in settings where routine health information is not available on a wide scale, is inconsistently collected or is unreliable [7,8]. DHS and MICS surveys interview caregivers / mothers of children and gather information on episodes of various childhood illnesses. These include suspected acute respiratory infections in children under five in the household and are based on a two week recall period. DHS and MICS surveys also gather information on reported use of antibiotics for these episodes [5,6]. Correct interpretation of surveys is needed to provide child health programmes with useful advice to guide activities. This in turn requires us to know the sensitivity and specificity of survey tools for detecting episodes of childhood disease. However, cross-sectional surveys are known to be prone to information error and bias with the potential for non-sampling errors from differing caregiver recall and survey instruments [9].

DHS and MICS surveys enquire about fever, cough, fast breathing with short, rapid breaths or difficulty breathing in the previous two weeks (and whether these were chest-related) [5,6]. DHS and MICS now label these as “symptoms of acute respiratory infection”. Despite this clear terminology, there are concerns that these episodes are often interpreted by other groups as a “proxy” for childhood pneumonia where only a minority of episodes reported represent true cases. One prospective study in Pakistan and Bangladesh found that current DHS and MICS methods discriminate poorly between children correctly diagnosed with pneumonia and those with cough/cold [10]. The positive predictive value of DHS and MICS tools has been estimated to be only 22% in identifying true pneumonia among those with reported symptoms of acute respiratory infection [11]. Despite these surveys enquiring about use of antibiotics for symptoms of acute respiratory infection, current survey tools may not provide an accurate denominator number of children with true pneumonia episodes to assess the percentage of children with pneumonia appropriately treated with antibiotics. Since the DHS and MICS survey data on antibiotic treatment relates to this group of children with ‘symptoms of acute respiratory infection’, there is a significant risk that any indicator measuring antibiotic treatment coverage for pneumonia may not be valid. Furthermore, concerns have been raised about the accuracy of caregiver recall of antibiotic use using current methods [10].

Population–based data on the proportion of children with pneumonia that are treated with an antibiotic is an important metric for district, national, regional and global monitoring of interventions designed to improve appropriate antibiotic treatment (and survival outcomes) for childhood pneumonia. However, if this metric is invalid it could lead to incorrect or uninformed programmatic decisions for the delivery of interventions. We conducted a prospective observational study to assess the validity of DHS5 and MICS5 questions in discerning true cases of pneumonia and treatment with antibiotics in the South-West region of Nigeria, a country with one of the highest numbers of child deaths from pneumonia in the world. We also aimed to assess differences in caregiver recall at two and eight week follow up and to test if caregiver recall of antibiotic treatment can be improved by use of an additional survey tool (a medicine pill board). Our results were compared to the results of a previous study (which investigated DHS and MICS survey tools in Asian countries) in order to provide recommendations on the appropriateness of the use of these metrics in the planning and monitoring of health programmes for tackling childhood pneumonia.

## METHODS

### Study sites

From 12 August 2015, patients were recruited from two sites: Out-patients Departments (OPD) of Oni Memorial Children’s Hospital, Ring Road and Adeoyo Maternity Teaching Hospital, Ibadan, Oyo State, Nigeria. These are the two biggest hospitals with the highest number of paediatric out-patients in this State. They are both secondary health facilities that serve patients from all areas of Ibadan with or without being referred. Ibadan is the third most populous city in Nigeria and was chosen as the study site given that no similar study had been conducted in an urban African city. When it became increasingly evident that the estimated sample size of 300 each of pneumonia and ‘no pneumonia’ cases would likely not be achieved within a proposed one year study period we enrolled cases from two additional hospitals; Our Lady of Apostle, Catholic Hospital, Oluyoro (a mission secondary health facility) and University College Hospital (a tertiary hospital with OPD) in Ibadan. This additional recruitment occurred from 14 April 2016 until the end of the study which was on 17 March 2017, giving a total recruitment period of 19 months.

### Study population

Children aged 0 to 59 months who present to the out-patient departments of the above hospitals suffering from cough and/ or difficult breathing and their mothers/caregivers.

### Study design

This was a prospective observational study to validate pneumonia diagnosis and recall of antibiotic treatment by caregivers. Two groups of children with acute respiratory symptoms - those who were confirmed to have pneumonia and those who did not have pneumonia according to WHO algorithms [11] for diagnosing clinical pneumonia - were identified, recruited and followed up at home. Clinical diagnosis of pneumonia by a study physician using the WHO clinical criteria for childhood pneumonia and verification of antibiotic prescriptions by study physicians were used as the gold standard for measuring the accuracy of DHS5 and MICS5 questionnaires [11,12]. Caregivers were surveyed to assess the accuracy of their recall of the diagnosis and treatment provided using DHS5 and MICS5 questionnaires. Caregivers were followed up after being randomly assigned to either two or eight weeks follow up after recruitment. Follow-up was done so as to assess whether increasing the length of the recall period from the two week recall period currently used in DHS and MICS surveys for symptoms of acute respiratory infection would decrease the accuracy or validity of caregiver report. This enabled an assessment to be made of the degree to which DHS and MICS measures of antibiotic treatment in those with reported symptoms of acute respiratory infection were valid measures of the antibiotic treatment of true pneumonia in a study population. Doctors, trained in the use of survey tools, interviewed the caregiver of each child in their home using DHS5 and MICS5 questionnaires and initially without, and then with a paper-based “medicine pill board”, which is a novel alternative tool using visual cues to assist in recalling antibiotic use. A WHO staff member with substantial experience in training for pneumonia diagnosis and management, Dr S.A. Qazi, visited the study sites on two occasions: first, before enrolment started and second, toward the middle of the study period on May 29 to June 3, 2016 to monitor the conduct of and progress with the study.

#### Sample size calculation

Based on baseline estimates of a sensitivity of 60%–70% and specificity of 70%–90% from a previous study [10] of mother/caregiver recall of symptoms of acute respiratory infection in predicting true pneumonia, it was estimated that 300 children under five years with physician-diagnosed pneumonia and 300 with “no pneumonia” should be enrolled in order to estimate sensitivity and specificity with a precision of +/− 5%. The primary caregivers who were the mothers of these children in virtually all the cases were first interviewed using the DHS5 and MICS5 questions relating to pneumonia either at two weeks (200 with pneumonia and 200 with cough or cold) or eight weeks (100 with pneumonia and 100 with cough or cold) after diagnosis.

#### Selection criteria

Inclusion criteria:

1. Children aged 0 to 59 months who present to the out-patient departments of the above hospitals suffering from cough and/or difficulty breathing and
2. Mother/ Caregiver willing to sign informed consent form.

Exclusion criteria:

1. Children presenting primarily with an episode of recurrent wheeze or asthma.
2. Children who have pneumonia with danger signs, severe enough to require hospital admission.
3. Children who have symptoms of more than 4 weeks’ duration.
4. Children who have previously come to the same health facility for treatment of the same illness episode and have already been enrolled in the study.
5. Children having history of recent pneumonia episode within past 10 days.
6. Children with history of congenital heart disease (suspected or confirmed).
7. Children who live outside Ibadan (or appropriate follow-up children living within the Ibadan Metropolis were enrolled).
8. Children whose mother/caregiver does not give consent.

### Enrolment and data collection

The sequence of recruitment of these children in the outpatient departments was as follows:

- Children were first registered at the OPD medical record section. All children aged 0-59 months were then referred to OPD.
- The regular OPD physicians examined and prescribed treatment to all these children. Children with cough and/or difficulty breathing were then referred to the research study physicians.
- The study physician then re-evaluated these children and confirmed that the child actually had pneumonia or not (according to the WHO guidelines on diagnosis of clinical pneumonia [12]). They then confirmed children matched the selection criteria.
- An informed written consent to participate was then taken from the caregivers.
- The study physician then proceeded with the enrolment. Baseline forms which included all their personal details were filled out for the recruited children.

#### Sampling technique

The follow-up sequence of two and eight weeks was administered randomly in a ratio 2:1. A computer generated randomized scheme was used for this purpose. The list was generated by the data manager and kept by the project manager. The project manager maintained a follow-up calendar and informed the study physicians of the follow-up schedule.

#### Case matching

Matching of pneumonia cases with no pneumonia cases was done using a procedure similar to that in a Bangladesh and Pakistan study [10]. Matching was performed after enrolment and follow-up of participants in the study. Pneumonia cases were matched with “no pneumonia” cases at the end of the study in order of preference as follows:

1. Matched on case’s sex, age (±2 months) and week of follow-up and assessed by the same study physician.
2. Matched on case’s sex, age (±2 months) and week of follow-up and assessed by different study physician.
3. Matched on case’s sex, age category (≤12 months or >12 months) and week of follow-up and assessed by the same or different study physician.

If more than one child with “no pneumonia” was found for a case (pneumonia), we randomly selected only one control using a Microsoft Excel generated random number.

#### DHS and MICS questionnaires and alternative measurement tools

DHS5 and MICS5 surveys both have an algorithm of questions about the presence or absence of specific signs and symptoms of “symptoms of acute respiratory infection” in DHS and MICS questionnaires [5,6] (Questions relating to ‘symptoms of acute respiratory infections’ have changed in subsequent iterations of DHS and MICS survey tools since DHS5 and MICS5). Questions ask about the presence of cough, faster breathing than usual, short, rapid breaths and difficulty breathing as well as whether caregivers thought difficulty breathing was due to a problem in the chest or to a blocked or runny nose. Questionnaires used during the study are included in Appendix S1 in **Online Supplementary Document(Online Supplementary Document)**.

The presence of fast breathing classifies patients as having ‘symptoms of acute respiratory infection’ in algorithms for DHS and MICS. These questions are similar but not identical with differences in wording and skip patterns between DHS5 and MICS5 [5,6].

An additional study tool using a drug chart prepared to document drugs which are commonly used in and around Ibadan to treat pneumonia, malaria and other common childhood febrile illnesses was employed. Pictures were also taken of the individual pills/syrups/injections available in the market, which were then loaded onto the study personal computer (PC) and printed in the form of a “medicine pill board” which the study physician carried during all house visits. Individual home follow-up interviews either at 2 weeks or 8 weeks were held with mothers/caregivers of children recruited from the hospital OPD. During these interviews, caregivers were initially asked about the use of drugs without showing the “medicine pill board” and then were shown the drug chart to assess: how well they recognized the drugs used for their children, whether the drugs included in the drug chart were used, and whether any additional drugs mentioned by the caregivers that were not included in the list of the “medicine pill board”.

### Statistical analysis

Descriptive statistics were used to assess the socio-demographic and clinical characteristics of the study children.

Sensitivity and specificity were calculated to assess the discriminative ability of each measurement tool (DHS5, MICS5, medicine pill board) that was used to reach a decision on the presence or absence of true pneumonia, with the area under the curve (AUC) of a receiver operating characteristic (ROC) curve presented as a summary of both sensitivity and specificity. Clinical diagnosis of pneumonia by a study physician using the WHO clinical criteria for childhood pneumonia and verification of antibiotic prescriptions by study physicians were used as the gold standard to compare with caregiver recall of pneumonia identification and antibiotic treatment. Positive predictive and negative predictive values are presented to illustrate the discriminative ability of the questionnaires to accurately determine pneumonia and “no pneumonia” respectively. Reported use of antibiotics with and without use of the ‘medicine pill board’ were then compared to the treatment recorded as given to calculate the sensitivity and specificity of questions in accurately recalling antibiotic use. The kappa statistic was used to assess the level of agreement between caregiver reports of antibiotic use and recorded treatment given. Associations in cross-tabulations are calculated with the χ^2^ test or, when expected counts are small, Fisher Exact test. All analyses were completed using STATA version 13 (Stata Corp, College Station, TX, USA), with the assumption of a *P* value <0.05 as statistically significant.

### Ethics statement

Ethical approval was obtained from the University of Ibadan/University College Hospital Ibadan Institutional Review Board, Oyo State Research Ethical Review Committee, Ministry of Health, Secretariat, Ibadan and the WHO Ethics Review Committee. We employed a two stage written consent procedure: caregivers were informed about the study and permission was obtained first at the time of diagnosis and enrolment from the hospital, and then again at the start of the follow-up home interviews.

## RESULTS

### Enrolment and follow-up

**Figure 1:**
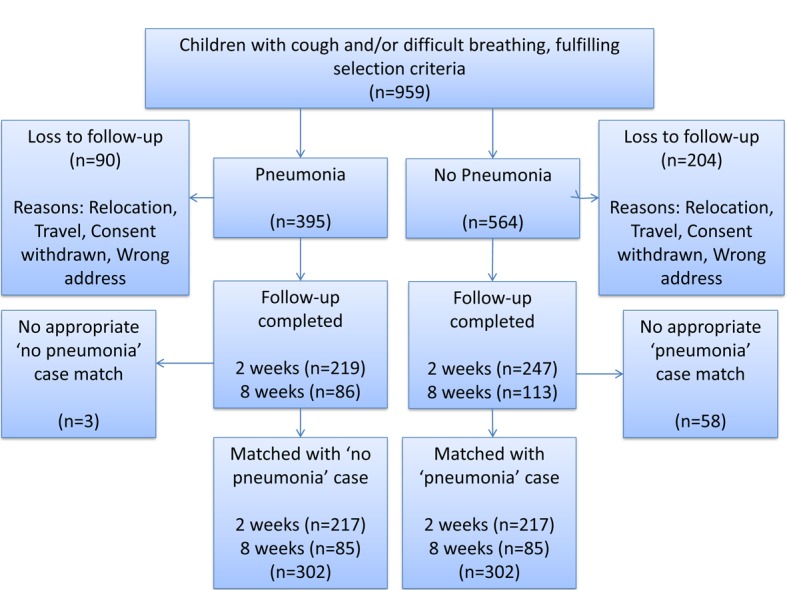
Enrolment and randomization of study patients.

From 12 August 2015 – 17 March 2017, 959 patients were enrolled with 665 patients completely followed-up. Of these patients, there were 305 with pneumonia, in which 219 and 86 had follow-up at two and eight weeks respectively. There were 360 with ‘’no pneumonia” (cough and/or difficult breathing) where 247 and 113 were followed-up at two and eight weeks respectively. However, three out of the 305 eligible and consented “pneumonia” patients were excluded from the database during matching as there were no “no pneumonia” patients that could match them based on age, sex and week of follow-up (**Figure 1**).

### Baseline characteristics of the study children

The socio-demographic characteristics and the clinical features of the matched 302 pneumonia cases and 302 cases without pneumonia are presented in **Table 1**. The socio-demographic characteristics of the two groups were similar. Expectedly, children with pneumonia had higher respiratory rates than those without pneumonia. Fever (axillary temperature ≥37.5°C) occurred more frequently in the pneumonia group than the “no pneumonia” group as did crepitations and bronchial breath sounds on chest auscultation (*P* < 0.001).

**Table 1:** Baseline characteristics of the study children*

Characteristics		Pneumonia (n = 302)		No pneumonia (n = 302)		*P*-value (χ^2^ test)
**Age of child (months) (mean ± SD)**		13.84 ± 12.06		15.92 ± 13.68		0.049
**Age category of child (months):**						
0 to 1		22 (7.28)		33 (10.93)		0.165
2 to 11		141 (46.69)		123 (40.73)		
12 to 59		139 (46.03)		146 (48.34)		
**Gender:**						
Male		168 (55.63)		168 (55.63)		1.000
Female		134 (44.37)		134 (44.37)		
**Siblings:**						
No siblings		95 (31.46)		109 (36.09)		0.228
One or more		207 (68.54)		193 (63.91)		
**Age category of mothers (years):**						
≤30		119 (39.40)		130 (43.05)		0.363
>30		183 (60.60)		172 (56.95)		
**Mother’s education:**						
None/primary		15 (4.97)		20 (6.62)		0.389
Secondary		110 (36.42)		96 (31.79)		
More than secondary		177 (58.61)		186 (61.59)		
**Father’s education:**						
None/Primary		9 (2.98)		8 (2.65)		0.421
Secondary		88 (29.14)		103 (34.11)		
More than secondary		205 (67.88)		191 (63.25)		
**Father’s occupation status:**						
Unemployed		7 (2.32)		7 (2.32)		1.000
Employed		295 (97.68)		295 (97.68)		
**Residence:**						
Rural		56 (18.54)		62 (20.53)		0.538
Urban		246 (81.46)		240 (79.47)		
**Symptoms:**						
Fever		194 (64.24)		144 (47.68)		<0.001
Cough and cold		301 (99.67)		299 (99.01)		0.624
Catarrh (running nose)		195 (64.57)		224 (74.17)		0.010
Respiratory problem†		29 (9.60)		15 (4.97)		0.028
Gastrointestinal upset‡		5 (1.66)		6 (1.99)		0.761
Diarrhoea		13 (4.30)		10 (3.31)		0.524
Vomiting		10 (3.31)		10 (3.31)		1.000
Other§		14 (4.64)		20 (6.62)		0.289
**Respiratory rate/min (mean ± SD):**						
0 to 1 months	59.95 ± 13.92		50.00 ± 6.42		0.007	
2 to 11 months	56.92 ± 9.25		40.95 ± 5.80		<0.001	
12 to 59 months	49.20 ± 8.80		33.26 ± 5.35		<0.001	
**Temperature (°C):**						
<37.5	213 (72.70)		243 (88.04)		<0.001	
≥37.5	80 (27.30)		33 (11.96)			
**Finding on auscultation:**						
No significant findings	201 (66.56)		298 (98.68)		<0.001	
Significant findings¶	101 (33.44)		4 (1.32)			

*Data collected by study physicians at enrolment.

†Respiratory problem – fast breathing; difficulty in breathing; nasal discharge; blocked nose; chest indrawing; grunting; sneezing.

‡Gastrointestinal upset – abdominal pain; poor appetite; poor feeding; oral sore.

§Other – body rash; abdominal rash; ear discharge; ear pain; recurrent boil; bilateral conjunctival redness.

¶Significant findings – crepitations; bronchial breath sounds; wheeze.

### Discriminative ability of survey tools

**Table 2** shows the sensitivity, specificity, 95% confidence intervals and Area Under the Curve (AUC) for ROC curves of the DHS5 and MICS5 tools studied. Data are expressed in percentages including confidence intervals.

**Table 2:** Discriminative power of DHS5/MICS5 questions for identifying childhood pneumonia (based on two and eight week recall)

Recall Period	Diagnostic validity	DHS5 Questions	Area under the curve*	MICS5 Questions	Area under the curve*
**2 weeks:**						
	**Sensitivity**	37.33 (30.87-44.13)	61.75 (57.79-65.71)	37.33 (30.87-44.13)	61.06 (57.04-65.08)
	**Specificity**	86.18 (80.86-90.47)		84.79 (79.31-89.29)	
**8 weeks:**						
	**Sensitivity**	36.47 (26.29-47.62)	63.53 (57.51-69.55)	36.47 (26.29-47.62)	62.94 (56.83-69.05)
	**Specificity**	90.59 (82.29-95.85)		89.41 (80.85-95.04)	
**Overall:†**						
	**Sensitivity**	37.08 (31.62-42.81)	62.25 (58.94-65.56)	37.09 (31.62-42.81)	61.59 (58.23-64.95)
	**Specificity**	87.42 (83.14-90.94)		86.09 (81.67-89.79)	

DHS - Demographic and Health Survey, MICS - Multiple Indicator Cluster Survey

*Receiver operator curve. Data are expressed as percentage with 95% confidence interval of percentage.

†Irrespective of the week of follow-up.

Results of the DHS5/MICS5 questions about suspected pneumonia for identifying childhood pneumonia shows the poor discriminative power of the survey tools. DHS5 and MICS5 results were similar, although their questions are not identical because of some differences in precise wording in the questionnaires and skip patterns. The discriminative power of these tools was not significantly different at two and eight week follow-up intervals as presented in **Table 2**.

### Antibiotic recall

92% of pneumonia cases and 84% of children without pneumonia were prescribed antibiotic treatment. Among caregivers whose child had pneumonia and were given antibiotics, correct treatment recall was 63.1% for DHS5 and 61.6% for MICS5 at two weeks follow up and 48.8% for DHS5 and 49.3% for MICS5 eight weeks follow-up respectively. Correct recall of antibiotic treatment increased to 88.8% and 82.3% using drug pillboard at two and eight weeks follow-up respectively (**Table 3**). Caregivers whose child had no pneumonia and were given antibiotics, correct treatment recall was (72.2%, 66.9%) and (64.4%, 56.9%) using DHS5 or MICS5 at two and eight weeks follow-up respectively. Correct recall of antibiotic treatment increased to 89.4% and 89.0% using drug pillboard at two and eight weeks follow-up respectively among this study group (**Table 3**). The specificity of recall in those who did not receive an antibiotic did decrease slightly when pill boards were used for both those with and without pneumonia. However, the overall level of agreement, as measured by the kappa statistic, indicates that pill boards improved correct recall of antibiotic treatment.

**Table 3:** Caregiver recall of antibiotic treatment*

Recall period	Tool	Pneumonia			No Pneumonia		
		**Sensitivity (95% CI)**	**Specificity (95% CI)**	**Kappa‡**	**Sensitivity (95% CI)**	**Specificity (95% CI)**	**Kappa‡**
**2 weeks:**							
	**DHS5**	63.13 (56.00-69.86)	61.11 (35.75-82.70)	0.090	72.22 (65.07-78.63)	72.97 (55.88-86.21)	0.316
	**MICS5**	61.63 (53.92-68.93)	66.67 (40.99-86.66)	0.114	66.87 (59.08-74.04)	82.35 (65.47-93.24)	0.316
	**Pill Board**	88.83 (83.58-92.87)	50.00 (26.02-73.98)	0.292	89.44 (84.01-93.52)	69.44 (51.89-83.65)	0.541
**8 weeks:**							
	**DHS5**	48.75 (37.41-60.19)	60.00 (14.66-94.73)	0.019	64.38 (52.31-75.25)	83.33 (51.59-97.91)	0.260
	**MICS5**	49.28 (37.02-61.59)	33.33 (0.84-90.57)	-0.028	56.94 (44.73-68.57)	90.00 (55.50-99.75)	0.114
	**Pill Board**	82.28 (72.06-89.96)	40.00 (5.27-85.34)	0.109	89.04 (79.54-95.15)	75.00 (42.81-94.51)	0.545
**Overall:†**							
	**DHS5**	58.99 (52.96-64.83)	60.87 (38.54-80.26)	0.064	69.96 (63.90-75.54)	75.51 (61.13-86.66)	0.298
	**MICS5**	58.09 (51.59-64.39)	61.90 (38.44-81.89)	0.066	63.83 (57.33-69.98)	84.09 (69.93-93.36)	0.279
	**Pill Board**	86.96 (82.40-90.69)	47.83 (26.82-69.41)	0.235	89.33 (84.85-92.85)	70.83 (55.94-83.05)	0.452

CI – confidence interval, DHS – Demographic and Health Survey, MICS – Multiple Indicator Cluster Survey

*Sensitivity and specificity values are expressed as percentage with 95% confidence interval of percentage.

†Irrespective of the week of follow-up.

‡Kappa refers to level of agreement between caregiver reports of antibiotic use through DHS or MICS tools or pill board with antibiotic treatment recorded at the hospital.

## DISCUSSION

DHS and MICS have a key role to play in providing population-based indicators to guide health programmes in countries with limited routine health data. However, few studies have evaluated the validity of DHS and MICS questions for evaluating antibiotic coverage for episodes of childhood pneumonia. This prospective observational study in South-West Nigeria adds to evidence from studies in Pakistan and Bangladesh [10] and finds similar conclusions in an African setting where malaria is an important child health problem and with high child mortality from pneumonia. DHS and MICS surveys are not designed to identify pneumonia cases and this study confirms the previous finding from the Asian studies that questions used in DHS5 and MICS5 surveys have poor sensitivity and specificity for discerning children who have pneumonia from those with no pneumonia. Thus, we suggest that this is a generally applicable finding which reflects the characteristics of the questions and the study populations of these surveys and is not related to specific study sites. The proportion of caregivers for children with pneumonia who had been treated with antibiotics, accurately recalled this only 58% and 59% of the time with MICS5 and DHS5 questions respectively. This recall improved to 87% with the use of a pill board and this should be considered as an inexpensive and feasible tool as part of DHS and MICS to improve accurate reporting of antibiotic use. Once again, this finding has now been replicated in several study sites and settings [10] and we suggest that this may be generally applicable.

**Figure 2:**
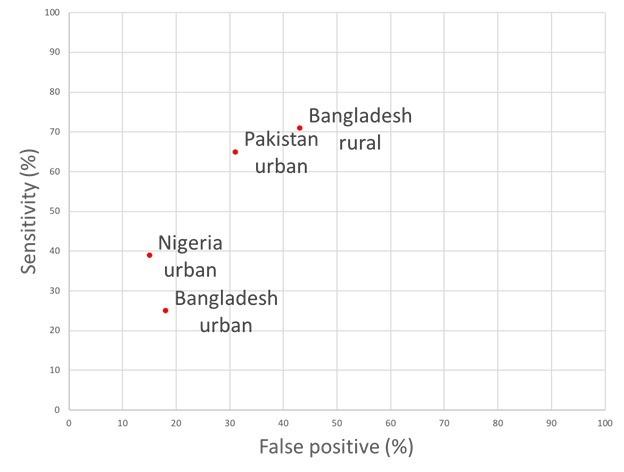
Receiver Operator Characteristic (ROC) curve of performance in discriminating children with pneumonia across four study sites, including Ibadan (Nigeria, urban); other data from [10,11].

Recent progress in improving access to treatment for childhood pneumonia remains a top priority for policy makers aiming to decrease preventable child deaths [4]. National programmes require appropriate evaluation measures to assess the prevalence of childhood pneumonia and to subsequently estimate the number of cases appropriately treated with antibiotics. Given the limitations of the population-based health information in most low-income countries and many lower-middle income countries, key policy decisions (especially those requiring population-based data) are often based on DHS and MICS surveys. However, on this particular metric, the proportion of children with pneumonia who received antibiotic treatment, DHS5 and MICS5 questions in this Nigerian study had poor sensitivity for identifying true pneumonia cases. Comparing this to study sites in Bangladesh and Pakistan, DHS and MICS questions in this study had significantly lower sensitivities for detecting true pneumonia (37.3, 95% CI = 30.9-44.1. (**Table 4**). The specificity of DHS5 and MICS5 questions in our study were 87.42 (83.14-90.94) and 86.09 (81.67-89.79) respectively. These were slightly better than those reported in study sites from Bangladesh and Pakistan (**Table 4**). However, the respective Area Under the Curve (AUC) estimates which summarise discriminative power of this approach in these different settings show that, although individual sensitivity and specificity values vary across sites, they all fall broadly in the same range of AUC values **(****Figure 2**). The reasons behind this substantial variation in sensitivity and specificity between countries and between study sites within countries are unclear and require further investigation. This level of unexplained variation is another major reason to urge caution in the use of these data. Differences in this indicator over time or across countries may be due to artefact based on variation in these underlying parameters rather than reflecting any change in antibiotic treatment practices. Estimates from these questions therefore should not be used to compare pneumonia prevalence or antibiotic treatment rates between study sites or countries.

**Table 4:** Specificity and sensitivity of DHS and MICS questions in Bangladesh, Pakistan and Nigeria study sites [10]

Study site (number of participants)	DHS sensitivity	MICS sensitivity	DHS specificity	MICS specificity
Bangladesh urban (n = 546)	24.6 (17.5-32.9)	25.4 (18.2-33.8)	81.7 (73.6-88.1)	82.5 (74.5-88.8)
Bangladesh rural (n = 455)	71.1 (61.0-79.9)	70.1 (60.0-79.0)	56.5 (45.3-67.2)	56.5 (45.3-67.2)
Pakistan urban (n = 672)	64.7 (58.4-70.9)	63.8 (57.5-70.0)	68.5 (62.5-74.4)	67.2 (61.1-73.2)
Nigeria urban (n = 604)	37.3 (30.9-44.1)	37.3 (30.9-44.1)	86.2 (80.9-90.5)	84.8 (79.3-89.3)
**Mean**	**49.4**	**49.2**	**73.2**	**72.8**

DHS – Demographic and Health Survey, MICS – Multiple Indicator Cluster Survey

The results of this study demonstrate that population-based survey questions to caregivers do not accurately discern true pneumonia cases from those with cough/cold (no pneumonia). This is not surprising given that we do not expect caregivers to be able to know when the symptoms in their child represent pneumonia and there is often unreliable and ineffective communication on diagnosis between health workers and caregivers. Although DHS and MICS report these data appropriately there is considerable potential, if reported as a proxy for childhood pneumonia, for these data to lead to incorrect global and local programme decisions. If we assume an underlying incidence of WHO defined pneumonia in developing countries of 0.22 per child per year [14] and an under five incidence of acute respiratory infection (ARI) in developing countries of 5.5-7.5 episodes per child per year [15-19] then several times more children who are identified by these questions about respiratory signs do not have pneumonia than have pneumonia. The recent changes to DHS and MICS questions (which adds specifically whether caregivers thought respiratory difficulties were ‘chest-related’) have led to the identification of a sub-group of more severely ill children with cough / cold as evidenced by the falling prevalence of these reports over time [20]. Nevertheless, children with pneumonia represent a minority of cases identified. These data should, therefore, not be used to estimate burden of pneumonia in young children [2] as they will over-estimate this substantially and are likely to vary significantly due to varying performance characteristics of this approach (as noted above). In addition, these survey questions currently do not provide an accurate denominator number of children with true pneumonia and so are severely limited in their ability to estimate appropriate antibiotic coverage for childhood pneumonia. Signs of acute respiratory infection is one of the commonest reasons for care seeking and for antibiotic treatment in young children [21]. Use of these data to estimate antibiotic treatment rates for child pneumonia can be expected to yield falsely low values and may lead to a drive within national programmes to promote and increase antibiotic prescription. This, in turn, could contribute to increasing overuse of antibiotics (due to inappropriate antibiotic prescribing for episodes of cough/cold) which underlies rising antimicrobial resistance globally.

We found that the use of pictorial representations of locally available treatments (a “medicine pill board”) improved accurate recall of antibiotic treatment. This was found consistently in both this study and other study sites in Pakistan and Bangladesh [10]. Poor antibiotic treatment recall without use of pill boards likely underestimates their usage. However, despite an increase in accuracy as measured by the Kappa statistic (**Table 3**), it is noted that there was a decrease in specificity of antibiotic recall. Therefore, further research into their use may be warranted but future surveys should consider incorporating similar visual cues and a number of DHS surveys have already incorporated their use. Pill boards have been shown to improve accurate recall of drugs in other settings [22].

Use of population-based indicators to measure national programme performance in tackling child pneumonia

Given current estimates of pneumonia incidence in developing countries of 0.22 episodes per child per year, two week recall surveys will not identify sufficient numbers of pneumonia cases to give accurate prevalence estimates unless very large numbers of children are surveyed [14]. This study did not find a significant difference in the specificity and sensitivity of survey questions between recall periods of two and eight weeks . Inaccurate reporting of childhood diseases may therefore not increase significantly when recall is increased by a short period of time [10,23]. Survey recall periods for episodes of acute respiratory infection could therefore be increased without losing quality of data and may capture more episodes in a single survey.

Health programmes should explore other avenues to collect health information on childhood pneumonia in low and lower-middle income countries. DHS and MICS may be able to provide sufficient information on care seeking rates for acute respiratory infections including pneumonia. Other studies could also investigate the quality of care from trained providers for childhood pneumonia and prevalence should also ideally be based on trained observation for signs of pneumonia and not maternal recall. Approaches such as these may provide more accurate measures for the management pathway of childhood pneumonia in future [24].

### 

#### Study limitations

This study is limited by the recruitment of children in hospital out-patient settings since those who sought care for their child’s illness may differ systematically from those who did not. Participants were also recruited from one area of Nigeria and, as has been seen in other studies, the performance of these questions can vary significantly by study site [10]. This may mean that the sample in this study is not truly generalisable to other children in other areas of Nigeria or, more generally, to low and lower-middle income countries. However, the main study conclusions do concur with the findings from study sites in Bangladesh and Pakistan. The recruitment of participants at the time of seeking care and alerting them to follow up may also have improved the recall of study participants when followed up subsequently. The same trained doctors who were involved in enrolment and diagnosis, also delivered these questions during follow up, which may have been a potential source of bias and the use of doctors to deliver questionnaires is different to the actual data collectors used in these surveys. There was loss to follow up which was higher in the ‘no pneumonia’ group as described in **Figure 1**. This is a potential source of bias to factor into interpretation of results. The specific wording of DHS and MICS questions have also been updated in the most recent iteration of surveys. Only a single assessor classified children as having pneumonia or not according to current WHO criteria which may have introduced bias when placing child in groups of with or without pneumonia. We attempted to mitigate this by arranging training and monitoring to be conducted by a highly experienced WHO staff member who had conducted the training in the previous study [10].
