# Supplementary material for: A prospective validation study in South-West Nigeria on caregiver report of childhood pneumonia and antibiotic treatment using Demographic and Health Survey (DHS) and Multiple Indicator Cluster Survey (MICS) questions
Source: J Glob Health. 2018 Sep 19;8(2):020806. doi: 10.7189/jogh.08-020806 (PMC6150611; doi:10.7189/jogh.08-020806)
Supplement: Online Supplementary Document [file jogh-08-020806-s001.pdf]

# Online Supplementary Document

**Ayede et al. A prospective validation study in South-West Nigeria on caregiver report of childhood pneumonia and antibiotic treatment using Demographic and Health Survey (DHS) and Multiple Indicator Cluster Survey (MICS) questions**

**J Glob Health 2018;8:020806**

## Appendix S1: DHS5 and MICS5 questionnaires used

## <u>DHS QUESTIONNAIRE</u>

533 *Has (NAME) been ill with a fever at any time in the last 2 weeks?*

| | |
|---|---|
| *Yes* | *1* |
| *No* | *2* |
| *Don't Know* | *8* |

*534* *Has (NAME) had an illness with a cough at any time in the last 2 weeks?*

| | |
|---|---|
| *Yes* | *1* |
| *No* | *2* |
| *Don't Know* | *8* |

*535* *When (NAME) had an illness with a cough, did he/she breathe faster than usual with short, rapid breaths or have difficulty breathing?*

| | |
|---|---|
| *Yes* | *1* |
| *No* | *2* |
| *Don't Know* | *8* |

*536* *Was the fast or difficult breathing due to a problem in the chest or to a blocked or runny nose?*

| | |
|---|---|
| *Chest Only* | *1* |
| *Nose Only* | *2* |
| *Both* | *3* |
| *Other* _______________ *(Specify)* | *4* |
| *Don't Know* | *8* |

*537* *Check 533: Had Fever?*

*538* *Now I would like to know how much (NAME) was given to drink (including breastmilk) during the illness with a (fever/cough). Was he/she given less than usual to drink, about the same amount, more than usual to drink?*
*If less, probe: Was he/she given much less than usual to drink or somewhat less?*

| | |
|---|---|
| *Much Less* | *1* |
| *Somewhat Less* | *2* |
| *About the Same* | *3* |
| *More* | *4* |
| *Nothing to Drink* | *5* |
| *Don't Know* | *8* |

539 *When (NAME) had a (fever/cough), was he/she given less than usual to eat, about the same amount, more than usual, or nothing to eat?*
*If less, probe: Was he/she given much less than usual to eat or somewhat less?*

| | |
|---|---|
| *Much Less* | *1* |
| *Somewhat Less* | *2* |
| *About the Same* | *3* |
| *More* | *4* |
| *Stopped Food* | *5* |
| *Never Gave Food* | *6* |
| *Don't Know* | *8* |

540 *Did you seek advice or treatment for the illness from any source?*

| | |
|---|---|
| *Yes* | *1* |
| *No* | *2* |

541 *Where did you seek advice or treatment? Anywhere else?*
*Probe to identify each type of source and circle the appropriate code(s).*
*If unable to determine if a hospital, health center or clinic is public or private medical, write the name of the place.*

________________________________
*(Name of place)*

***Public Sector***

| | |
|---|---|
| *Govt Hospital* | *A* |
| *Govt Health Center* | *B* |
| *Govt Health Post* | *C* |
| *Mobile Clinic* | *D* |
| *Fieldworker* | *E* |
| *Other Public* | *F* |

_________________ *(specify)*

***Private Medical Sector***

| | |
|---|---|
| *PVT Hospital/Clinic* | *G* |
| *Pharmacy* | *H* |
| *PVT Doctor* | *I* |
| *Mobile Clinic* | *J* |
| *Fieldworker* | *K* |
| *Other Private Med.* | *L* |

_________________ *(specify)*

***Other Source***

| | |
|---|---|
| *Shop* | *M* |
| *Traditional Practitioner* | *N* |
| *Other* | *X* |
| ________________ *(specify)* | |

*543 Where did you first seek treatment? Use letter code from 541.*

*544 How many days after the illness began did you first seek advice or treatment for (NAME)? If the same day, record '00'.*

*545 Is (NAME) still sick with (fever/cough)?*

| | |
|---|---|
| *Fever Only* | *1* |
| *Cough Only* | *2* |
| *Both Fever and Cough* | *3* |
| *No, Neither* | *4* |
| *Don't Know* | *8* |

*546 At any time during the illness, did (NAME) take any drugs for the illness?*

| | |
|---|---|
| *Yes* | *1* |
| *No* | *2* |

*547 What drugs did (NAME) take? Any other drugs? Record all mentioned.*

| | |
|---|---|
| *Antimalarial Drugs* | |
| *SP/Fansidar* | *A* |
| *Chloroquine* | *B* |
| *Amodiaquine* | *C* |
| *Quinine* | *D* |
| *Artemeter / Lumefantrin* | *E* |
| *Atesunate / Amodiaquine* | *F* |
| *Other Antimalarial* | *G* |
| ________________ *(specify)* | |
| *Antibiotic Drugs* | |
| *Pill/Syrup* | *H* |
| *Injection* | *I* |
| *Specify Name of Antibiotic* ________________ | |
| *Other Drugs* | |
| *Aspirin* | *J* |
| *Acetaminophen* | *K* |
| *Ibuprofen* | *L* |
| *Other* ________________ *(specify)* | *X* |
| *Don't Know* | *Z* |

*Followed by specific questions about each drug mentioned…*

## MICS QUESTIONNAIRE

| | | |
|---|---|---|
| CA7. AT ANY TIME IN THE LAST TWO WEEKS, HAS (*name*) HAD AN ILLNESS WITH A COUGH? | Yes ........ 1<br>No ........ 2<br><br>DK ........ 8 | <br>2⇨CA14<br><br>8⇨CA14 |
| CA8. WHEN (*name*) HAD AN ILLNESS WITH A COUGH, DID HE/SHE BREATHE FASTER THAN USUAL WITH SHORT, RAPID BREATHS OR HAVE DIFFICULTY BREATHING? | Yes ........ 1<br>No ........ 2<br><br>DK ........ 8 | <br>2⇨CA14<br><br>8⇨CA14 |
| CA9. WAS THE FAST OR DIFFICULT BREATHING DUE TO A PROBLEM IN THE CHEST OR A BLOCKED OR RUNNY NOSE? | Problem in chest ........ 1<br>Blocked or runny nose ........ 2<br><br>Both ........ 3<br><br>Other (*specify*)__________ 4<br>DK ........ 8 | <br>2⇨CA14<br><br><br><br>6⇨CA14 |
| CA10. DID YOU SEEK ANY ADVICE OR TREATMENT FOR THE ILLNESS FROM ANY SOURCE? | Yes ........ 1<br>No ........ 2<br><br>DK ........ 8 | <br>2⇨CA12<br><br>8⇨CA12 |
| CA11. FROM WHERE DID YOU SEEK ADVICE OR TREATMENT? | | |
| CA12. WAS (NAME) GIVEN ANY MEDICINE TO TREAT THIS ILLNESS? | Yes ........ 1<br>No ........ 2<br><br>DK ........ 8 | <br>2⇨CA14<br><br>8⇨CA14 |
| CA13. WHAT MEDICINE WAS (NAME) GIVEN?<br><br>PROBE:<br>ANY OTHER MEDICINE?<br><br>CIRCLE ALL MEDICINES GIVEN. WRITE BRAND NAME(S) OF ALL MEDICINES MENTIONED.<br><br><br><br>(NAMES OF MEDICINES) | Antibiotic<br>Pill / Syrup ........ A<br>Injection ........ B<br><br>Anti-malarials ........ M<br><br>Paracetamol / Panadol / AcetaminophenP<br>Aspirin ........ Q<br>Ibuprofen ........ R<br><br>Other (specify) ........ X<br>DK ........ Z | |
